# The mental health crisis in global higher education: understanding and mitigating academic load stress among international students from Asia and Africa in Nanjing China

**DOI:** 10.3389/fpsyg.2026.1707944

**Published:** 2026-01-22

**Authors:** Wang Suyuhan, Abdul Rasool Khoso, Gu Jintu, Shahnaz Bhutto

**Affiliations:** Department of Sociology, School of Public Administration, Hohai University Nanjing, Nanjing, China

**Keywords:** academic stress, higher education, international students, Nanjing, mental health, China

## Abstract

The mental health challenges faced by international students in higher education institutions worldwide have reached critical levels, with academic stress identified as a primary contributing factor. This study investigates the psychological effects of academic load stress on international students in Nanjing, China, emphasizing the complex interaction between cultural, academic, and social stressors. Employing a mixed-methods design, the research integrates quantitative surveys (*n* = 1,115) with qualitative interviews (*n* = 10) to analyze stress triggers, mental health outcomes, and institutional support mechanisms. Key results indicate 40% of participants experienced frequent stress symptoms. Hierarchical regression analysis identified academic workload as the strongest predictor for stress (*β* = 0.37, *p* < 0.001), anxiety (*β* = 0.33, *p* < 0.001), and depression (*β* = 0.29, *p* < 0.001), with the full model explaining a significant portion of variance (Total *R*^2^ = 0.154 for stress, Δ*R*^2^ = 0.102 for academic factors). Social isolation and language barriers were also significant contributors across all mental health outcomes. Stress levels varied by program, with Master’s students reporting the highest prevalence of moderate stress (48%) and PhD candidates the highest severe stress (45%). Qualitative data revealed cultural adaptation difficulties, including challenges with Confucian educational norms and language proficiency, which intensified stress. Despite existing support services, only 38% of students utilized counseling, citing stigma and cultural misalignment as barriers. This study highlights the urgent need for tailored interventions, such as culturally responsive faculty training, peer support networks, and improved mental health resources. By contextualizing global research within China’s higher education landscape, the findings advance understanding of academic stress and propose practical strategies to enhance student well-being in international academic environments.

## 1 Introduction

### 1.1 Background of the research

The number of International students in China has increased, with more than 492,000 enrolled in 2018 (China, 2018-2019; Ying et al., 2025), while studying globally can be enlivening it also poses challenges as students adjust to a new cultural environment. The rising prevalence of mental health challenges among university students has become a critical issue in global higher education, with academic stress being a leading contributor (Ochanda, 2024). This large-scale, global study of students from 21 countries provides powerful epidemiological evidence for the worldwide scale of the problem, showing that one in three students reports a mental health disorder (Lucas, 2009). It establishes the “critical issue” claim (Acevedo, 2023). This alarming prevalence firmly establishes student mental health as a critical priority for higher education institutions globally (Bantjes et al., 2022). Academic stress, in particular, has emerged as a leading contributor to psychological distress, affecting students’ overall well-being and academic performance (Ansari et al., 2024). The construct of academic stress encompasses chronic pressures from high evaluative expectations, excessive workload, and intense competition, which have been consistently linked to heightened levels of anxiety, depression, and burnout in student populations (Pascoe et al., 2020). The pathogenic role of academic pressure is further supported by longitudinal evidence, demonstrating that increases in academic demands predict subsequent declines in psychological well-being and engagement (Datu and King, 2018).

The pressure to excel in a highly competitive environment, coupled with financial burdens and social expectations, has created a perfect storm for mental health challenges (Ning et al., 2024; Zhang et al., 2024). This confluence of stressors represents a syndemic, where multiple adversities interact synergistically to worsen outcomes. Financial precarity, for instance, is not merely a background worry but an active stressor that forces students into difficult work-study balances, reduces time for recovery, and amplifies the perceived stakes of academic failure (Richardson et al., 2017). Simultaneously, social and familial expectations often internalized as a fear of disappointing others or losing status compound performance anxiety, creating a state of chronic hyper-arousal that depletes coping resources (Conley et al., 2020). International students are particularly vulnerable due to the compounded pressures of cultural adaptation, language barriers, and rigorous academic demands (Ying et al., 2024). While this issue is widespread across all student populations, international students face unique and compounded stressors that make them particularly vulnerable. Their experience is fundamentally framed by the acculturation process, a major life transition that involves navigating unfamiliar social norms, values, and daily practices. Berry’s model of acculturative stress provides a key theoretical lens, positing that the strain of adapting to a new culture, especially when coupled with experiences of perceived discrimination or social exclusion, constitutes a significant risk factor for mental health disorders (Berry, 2006). In China, where universities are increasingly attracting a diverse student body, cities such as Nanjing have seen a surge in international enrollments. These students must adjust not only to a new educational system but also to societal expectations that may differ significantly from their home countries (Nirmalan et al., 2025). The Chinese higher education context, with its distinct emphasis on examination performance, hierarchical teacher-student dynamics, and collective academic achievement, presents a specific acculturative challenge (Wang and Ma, 2022). International students may struggle to reconcile these expectations with the more individualistic or participatory educational paradigms from their home cultures. Additionally, language barriers, cultural adoption and the academic pressure has been the major concern in Chinese universities for the international students (Hussain and Shen, 2019). The resulting stress from academic workload poses serious risks to their psychological well-being, academic success, and long-term retention (Chen and Khoso, 2025). Therefore, investigating the specific mechanisms through which academic and acculturative stress interact for international students in contexts like China is not only academically pertinent but also crucial for developing inclusive, effective support frameworks that can safeguard student well-being and ensure the success of globalized higher education initiatives.

Numerous studies highlight that stigma remains a significant barrier to mental health help-seeking among university students globally, with particularly pronounced effects in Chinese cultural contexts due to collectivist values emphasizing family reputation (mianzi) and the perception of psychological distress as a weakness of character or a somatic issue rather than a legitimate health concern (Al Atoum, 2025; Chen et al., 2024); research indicates that students, especially males, often internalize these stigmatizing beliefs, leading to self-stigma and a preference for informal support networks over professional counselling due to fears of social exposure, doubts about efficacy, and a desire to handle problems autonomously to avoid burdening others (Gulliver et al., 2010), underscoring a critical need for culturally adapted interventions that address these deep-seated attitudinal and systemic barriers within educational environments (Xu et al., 2025).

### 1.2 Case studies from different nations

Research in Spanish highlights academic stress among international students due to unfamiliar teaching methods and high expectations, as seen in Erasmus programs where differing assessment styles such as a sudden shift from continuous coursework to a single final exam caused anxiety (Idoiaga-Mondragon et al., 2025; Köylü and Bulut-Şahin, 2024) and in the UK where intensive coursework led to overwhelm (Adisa et al., 2019). Similarly, in Japan, students face stress from self-directed learning and hierarchical classroom norms (Suenaga, 2025), where students may hesitate to ask clarifying questions for fear of disturbing the social order (uchi/soto), can lead to isolation and confusion. While in South Korea, competitive STEM programs and perfectionism contribute to anxiety (Kwon and Cho, 2020), fueled by a culture of perfectionism and high-stakes exams, is strongly correlated with anxiety and burnout, often manifesting as a fear of “face” loss (chemyon) for the student and family. In France, theoretical teaching and competitive grading in Grandes Écoles create challenges (Borredon et al., 2011), the emphasis on highly abstract, theoretical teaching (e.g., intense focus on mathematical proofs and philosophical concepts in preparatory classes) often prioritizes intellectual brilliance over applied knowledge, creating a steep learning curve. These strategies, while sometimes enabling short-term survival, significantly increase vulnerability to chronic anxiety, depressive episodes, and emotional exhaustion (Gonçalves and Matos, 2025). Whereas Germany’s research-focused system, with its emphasis on autonomy and final exams, adds pressure (Amos et al., 2008). These findings reveal that while academic stress is universal, its manifestations vary based on institutional and cultural contexts.

### 1.3 Research gap

In China, academic culture is deeply influenced by Confucian values, which prioritize diligence, respect for authority, and high achievement (Du and Li, 2024). This cultural foundation shapes a distinct pedagogical ecosystem characterized by high-contact teaching hours, a strong emphasis on rote memorization and technical mastery, and an assessment system heavily reliant on high-stakes, summative examinations (Jin and Cortazzi, 2006). For international students, this can translate into additional stress as they navigate expectations that may contrast with their previous educational experiences. However, there is limited research exploring how these cultural and academic pressures specifically affect international students in Nanjing. European higher education systems (e.g., the Bologna Process-influenced models) often emphasize student-led inquiry, critical analysis, continuous assessment (e.g., essays, projects), and fewer mandatory contact hours, fostering greater independent learning (Mok, 2016). Conversely, many African university systems, while diverse, may share a legacy of exam-centric assessment but often with vastly different resource constraints and student-instructor ratios (Assié-Lumumba, 2011). By examining case studies from Europe alongside the unique dynamics of Chinese higher education, this study aims to identify key stressors and propose targeted interventions to alleviate academic load stress. Applying a behavioral science perspective, this research seeks to enhance institutional support systems, mental health resources, and teaching strategies that promote student resilience. By bridging insights from European research with the realities of studying in China, this paper contributes to a more comprehensive understanding of student well-being in transnational education.

### 1.4 Theoretical framework

This study is anchored in an integrated theoretical model that combines Job Demands–Resources (JD-R) theory (Bakker and Demerouti, 2017), with Lazarus and Folkman’s Transactional Model of Stress and Coping (Lazarus and Folkman, 1987), situated within the context of acculturative and minority stress frameworks (Berry, 2006). We conceptualize academic workload, language barriers, and research demands as primary demands that deplete psychological resources, while institutional support and social integration function as critical resources that buffer against stress. The pathway from these demands to mental health outcomes (stress, anxiety, depression) is theorized to operate through mechanisms of chronic resource depletion (Hobfoll, 1989) and failed coping transactions, particularly under conditions of high acculturative pressure. For international students in China, Confucian educational norms and collectivist social expectations represent culturally-specific demands that intersect with academic pressures (Pérez-Jorge et al., 2025), exacerbating perceived stress and limiting help-seeking behaviors due to stigma (Yang, 2011). This framework informs our analytical strategy: hierarchical regression will test the additive contribution of academic, social, and cultural demands to stress variance, while correlation and qualitative analyses will explore mediating pathways (e.g., social isolation as a mediator between language barriers and depression) and moderating effects (e.g., degree level as a moderator of workload impact).

## 2 Research methodology

### 2.1 Research design

This study adopted a mixed-methods approach, combining quantitative surveys with qualitative interviews to comprehensively examine academic stress and mental health among international students in Nanjing, China. This method is mainly used in Social sciences research (Creswell, 1999). The explanatory sequential design allowed for an initial quantitative phase to identify stress patterns, followed by in-depth qualitative interviews to explore underlying causes and lived experiences (Ivankova et al., 2006). This integration provided a richer understanding of the data while maintaining methodological rigor.

### 2.2 Study area and sample size

The research was conducted in different universities of Nanjing including (Southeast university, Hohai university, Nanjing Normal university and Nanjing University), a major educational hub hosting a diverse international student population. The quantitative sample included 1,115 students from undergraduate, master, and PhD programs from African and Asian students because of the growing numbers of the students, ensuring sufficient statistical power for regression and correlation analyses. Of the 1,500 surveys distributed, 1,115 were returned, resulting in a response rate of 74.3%. Nonresponse was addressed through in-person follow-ups, but no formal analysis of nonrespondents was conducted, which may introduce some selection bias toward more accessible or motivated students. For the qualitative phase, 10 participants were purposively selected from survey respondents to ensure diversity across key dimensions: degree program (4 Master’s, 4 PhD, 2 Undergraduate), region of origin (6 Asia, 4 Africa), and gender (6 male, 4 female). The primary selection criterion was variation in self-reported stress levels (low, moderate, high) to capture a wide spectrum of experiences. The data was collected in different times January-2024 to October-2024 due to the availability of the students.

### 2.3 Sampling method

For quantitative phase, a stratified random sampling technique was employed to ensure proportional representation across degree levels and geographic regions (Hossan et al., 2023). The quantitative sampling frame consisted of the official enrollment lists of international students from the four participating universities in Nanjing (Southeast University, Hohai University, Nanjing Normal University, Nanjing University) for the 2023/2024 academic year. A stratified random sampling technique was employed. The population was first stratified by university (4 strata) and then by degree level (Undergraduate, Master’s, PhD) within each university. Students from Africa and Asia were specifically targeted within these strata due to their predominance in the international student population at these institutions, aligning with the study’s focus on the largest demographic groups. Structured questionnaires were administered to collect quantitative data, enabling numerical analysis of key variables. For qualitative phase, purposive sampling was utilized to select interview participants based on survey responses, ensuring the inclusion of key demographic subgroups and varying stress levels using in-depth interviews. This approach facilitated in-depth exploration of behavioral patterns and subjective experiences.

### 2.4 Data collection

The quantitative component utilized validated scales to operationalize key constructs: perceived stress was measured using a 5-point Likert scale (anchored from 1 = strongly disagree to 5 = strongly agree), since, the five point Likert scale is purposively used to conduct the subjective perceptions, attitudes, and levels of agreement regarding academic and acculturative stressors, aligning with its validated use in perception-based educational research (Joshi et al., 2015), This format was chosen for its reliability, ease of comprehension across a linguistically diverse sample, and proven effectiveness in measuring attitudinal constructs central to this study. While mental health symptoms were assessed through self-reported frequency of stress, anxiety, and depressive symptoms (categorized as never, sometimes, often, or always). This categorical scale was selected to efficiently capture the spectrum of everyday psychological distress that impacts academic functioning, which is a primary focus of this investigation, rather than clinical diagnosis. Academic stressors were evaluated across three domains - research demands, coursework load, and language difficulties - each rated on a 5-point intensity scale. Social determinants included Likert-scale measures of isolation and language barriers. Demographic covariates captured gender, age, geographic region, and academic level. The qualitative investigation employed in-depth, semi-structured interviews (mean duration = 38 min) to elicit rich narratives about: (1) lived experiences of academic pressure, (2) adaptive and maladaptive coping strategies, (3) perceived adequacy of institutional resources, and (4) acculturative stress processes. Sample questions included: “*Can you describe a typical week in terms of your academic workload and how you manage it?”; “In what situations do you feel the most stress or isolation, and how do you respond?”; and “What has been your experience, if any, with seeking support from the university for stress or mental health concerns*?” Semi structured In-depth interviews are accepted in social science research for exploring complex phenomenological realities (Bryman, 2016). This method was specifically chosen for its capacity to generate detailed, contextualized understanding of how the stressors quantified in the survey are experienced, interpreted, and navigated by students in their own words, thereby uncovering the mechanisms behind the statistical relationships. All interviews were digitally recorded, professionally transcribed, and subjected to rigorous translation protocols for non-native English speakers, with particular attention to maintaining semantic fidelity and cultural context during linguistic conversion. To ensure participants could express themselves with maximal comfort and nuance, interviews were conducted in the participant’s language of choice including English and Urdu. This multilingual approach was central to our commitment to cultural and linguistic sensitivity, aiming to reduce measurement error caused by language barriers. This protocol was implemented to maximize construct validity by allowing participants to articulate complex emotional and experiential nuances in their preferred language, thereby ensuring the data captured a more authentic representation of their lived reality. This mixed-method design enabled both psychometric quantification of stress correlates and phenomenological examination of distress experiences.

### 2.5 Data analysis

Quantitative analysis employed descriptive statistics (frequencies, percentages, means) to summarize demographic and stress distributions (Tables 1–3), followed by Hierarchical linear regression was performed in three blocks (demographics, academic factors, social factors) to identify stress predictors, with results reported via *β* coefficients, *p*-values (FDR-adjusted), 95% CIs, and ΔR^2^ (Table 4). Pearson’s correlations (*r*) (Cleophas et al., 2018) examined associations between stressors and mental health outcomes also FDR corrected (Table 5). For qualitative analysis, interview transcripts underwent (Braun and Clarke, 2006) thematic analysis. To ensure analytic rigor and auditability, we implemented a structured consensual coding strategy. Two researchers independently coded three randomly selected transcripts (30% of the sample) to develop an initial codebook. Discrepancies were discussed until full consensus was reached, refining the code definitions. This consensual approach, rather than calculating inter-coder reliability (*κ*), was chosen for its alignment with the interpretive, constructivist paradigm of our study, where consensus through discussion enhances the conceptual depth and validity of the themes (Braun and Clarke, 2006). The final codebook was then applied to the remaining transcripts by the primary researcher, with weekly peer debriefing sessions to discuss emerging themes and ensure consistency. Saturation was assessed during the coding process. Data collection and analysis were concurrent for the qualitative phase. We determined that thematic saturation was achieved after the 8th interview, as two subsequent interviews yielded no new codes or substantive insights into the established thematic framework, only reinforcing existing patterns. After transcription and iterative review, inductive coding identified emergent patterns (e.g., “workload overload,” “social isolation”), which were refined into broader themes (e.g., “cultural barriers to institutional support”). Finally, the thematic analysis was done using manually through the process of examining the themes, subthemes and interpreted accordingly in the results.

**Table 1:** Confirmatory factor analysis model fit indices.

Model	Overall CFA model
χ^2^(df)	685.42 (293)
P	<0.001
CFI	0.94
TLI	0.93
RMSEA [90% CI]	0.055 [0.050–0.060]
SRMR	0.048

**Table 2:** Demographic characteristics of international students in Nanjing (*N* = 1,115).

Variable	Category	Frequency (n)	Percentage (%)
Gender	Male	624	56.0
	Female	491	44.0
Age	20–25	298	26.7
	26–30	439	39.4
	31–40	378	33.9
Region	Africa	468	41.9
	Asia	647	58.1
Degree Level	Undergraduate	223	20.0
	Master’s	502	45.0
	PhD	390	35.0

**Table 3:** Prevalence of mental health symptoms.

Symptom	Never (%)	Sometimes (%)	Often (%)	Always (%)
Stress	12.0	48.0	32.0	8.0
Anxiety	18.0	42.0	28.0	12.0
Depression	22.0	38.0	27.0	13.0

**Table 4:** Hierarchical regression analyses predicting stress, anxiety, and depression among international students in Nanjing (N = 1,115).

Predictor	Stress	Anxiety	Depression
	*β* [95% CI] (FDR-adj *p*)	*β* [95% CI] (FDR-adj *p*)	*β* [95% CI] (FDR-adj *p*)
Step 1: demographics			
Female (ref: Male)	0.18 [0.04, 0.32] (0.015)	0.22 [0.08, 0.36] (0.008)	0.20 [0.06, 0.34] (0.011)
Age	−0.07 [−0.15, 0.01] (0.096)	−0.05 [−0.13, 0.03] (0.148)	−0.08 [−0.16, 0.00] (0.086)
Step 1 *R*^2^/ΔR^2^	0.017/0.017	0.021/0.021	0.019/0.019
Step 2: academic			
Master’s	0.16 [0.08, 0.40] (0.090)	0.14 [0.06, 0.38] (0.112)	0.12 [0.04, 0.36] (0.136)
PhD	0.24 [−0.02, 0.34] (0.009)	0.26 [−0.01, 0.36] (0.007)	0.28 [0.01, 0.38] (0.005)
Academic Load	0.37 [0.25, 0.49] (<0.001)	0.34 [0.22, 0.46] (0.001)	0.31 [0.19, 0.43] (0.003)
Step 2 *R*^2^/Δ*R*^2^	0.119/0.102	0.128/0.107	0.122/0.103
Step 3: social			
Social isolation	0.22 [0.08, 0.36] (0.006)	0.19 [0.05, 0.33] (0.015)	0.25 [0.11, 0.39] (0.004)
Language barrier	0.15 [0.03, 0.27] (0.020)	0.17 [0.05, 0.29] (0.012)	0.14 [0.02, 0.26] (0.024)
Step 3 *R*^2^/Δ*R*^2^	0.154/0.035	0.152/0.024	0.157/0.035
Total model *R*^2^	**0.154**	**0.152**	**0.157**

β = standardized beta coefficient; CI = Confidence Interval; *R*^2^ = variance explained; Δ*R*^2^ = change in *R*^2^ from previous step. All *p*-values are adjusted using False Discovery Rate (FDR) correction (Benjamini and Hochberg, 1995) across all three models (21 tests total).

**Table 5:** Correlation matrix of stressors and mental health.

Variable	1	2	3	4	5	6
1. Academic load	1.00					
2. Language barrier	0.43**	1.00				
3. Social isolation	0.31**	0.27**	1.00			
4. Stress	0.58**	0.42**	0.34**	1.00		
5. Anxiety	0.53**	0.39**	0.29**	0.68**	1.00	
6. Depression	0.51**	0.36**	0.32**	0.65**	0.72**	1.00

*p* < 0.01 (FDR-corrected). All correlations are Pearson’s *r* coefficients.

### 2.6 Ethical considerations and informed consent

Ethical review for the research “The Mental Health Crisis in Global Higher Education: Understanding and Mitigating Academic Load Stress among International Students in Nanjing China” was waived by the Ethics Review Committee of the School of Public Administration, Hohai University (Reference: 20250301). The Committee determined that the study qualified for exemption based on the following factors: the study did not involve vulnerable populations or minors, posed no foreseeable risks to participants, and involved standard survey and interview procedures where participation was voluntary and anonymous. The study adhered to the ethical standards outlined in the Declaration of Helsinki (1964) and its subsequent amendments. Informed consent was obtained from all participants, who were fully informed about the research aims and procedures. Participants were assured of confidentiality regarding their personal information and were free to withdraw at any time without penalty. All participants indicated their satisfaction with the procedures.

### 2.7 Validity and reliability of the data

Validity and reliability are critical for minimizing measurement errors and ensuring the robustness of research instruments. Validity assesses whether a tool measures what it intends to measure, while reliability evaluates the consistency of the results over time or across items. In this study, reliability was tested using Cronbach’s alpha (*α* ≥ 0.7), confirming strong internal consistency for scales measuring mental health symptoms, academic stress, and support services. Confirmatory Factor Analysis (CFA) further validated the constructs, with factor loadings and Average Variance Extracted (AVE) values exceeding 0.40, meeting established thresholds for convergent validity as shown in Table 6. The Composite Reliability (CR) for all constructs was above 0.70, indicating satisfactory reliability. Detailed item-level factor loadings, AVE, and CR values are presented in Table 6. These analyses demonstrate that the instruments were both reliable and valid for assessing the target variables.

**Table 6:** Validity and reliability in study instruments.

Appendix-I	Variable	Items	Cronbach’s alpha		CFA
			Pre-test (*n* = 35)	Final-test (*n* = 1,115)	AVE≥0.40
Reliability	Anxiety	7	0.81	0.73	0.45
	Academic stress	7	0.84	0.74	0.53
	Depression	7	0.78	0.71	0.51
	Support service	3	0.79	0.75	0.56

To establish the psychometric robustness of our measurement model, we conducted a comprehensive confirmatory factor analysis (CFA) for the four latent constructs Anxiety, Academic Stress, Depression, and Support Services specified as reflective and allowed to correlate. Model fit was assessed using multiple indices, with the following thresholds applied: Comparative Fit Index (CFI) and Tucker–Lewis Index (TLI) values ≥ 0.90 indicating acceptable fit and ≥ 0.95 good fit (Hu and Bentler, 1999), Root Mean Square Error of Approximation (RMSEA) values ≤ 0.08 acceptable and ≤ 0.06 good fit (MacCallum et al., 1996), and Standardized Root Mean Square Residual (SRMR) values ≤ 0.08 acceptable (Hu and Bentler, 1999). The overall CFA model demonstrated good fit to the data (see Table 1), and all standardized factor loadings were statistically significant (*p* < 0.001) and exceeded the recommended threshold of 0.50 (range: 0.58–0.86), supporting convergent validity at the item level (see Supplementary Table S1 for full loadings).

## 3 Results

The data in Table 2 examined stress and mental health among 1,115 international students in Nanjing, revealing critical insights into demographic, academic, and social factors influencing psychological well-being. The sample comprised slightly more males (56%) than females (44%), with the largest age group being 26–30 years (39.4%). Geographically, Asian students (58.1%) formed the majority, followed by African students (41.9%). Academically, master’s students were the largest cohort (45%), indicating a population where graduate-level academic pressures may be a significant factor. These demographic trends align with prior research suggesting that gender, age, and academic level play key roles in stress adaptation among international students.

Mental health symptoms were prevalent, with 40% of students reporting frequent stress (“often” or “always”), and similar rates observed for anxiety and depression as shown in Table 3. This high prevalence suggests that international students face substantial psychological challenges, possibly due to acculturative stress, academic demands, or social isolation. The findings underscore the need for proactive mental health interventions in academic institutions hosting large numbers of international students.

### 3.1 Pearson χ^2^ test of independence

Regional differences in stress levels were notable in Table 7. African students reported higher moderate stress (51.9%), whereas Asian students experienced more high stress (40.1%). This divergence may reflect cultural coping mechanisms, differing academic expectations, or variations in social support networks. For instance, Asian students, often from highly competitive educational backgrounds, might face intensified pressure to excel, while African students could encounter transitional stress related to cultural adjustment. The Table 3 presents stress level distributions among international students across different academic programs, revealing distinct patterns. Master’s students exhibit the highest stress levels, with 37% reporting high stress significantly more than undergraduates (25%) and PhD candidates (45%) while only 15% experience low stress, suggesting transitional challenges in this phase. PhD students show a polarized distribution, with the largest high-stress group (45%) but also a notable low-stress cohort (20%), possibly reflecting adaptation over time. Undergraduates demonstrate the most balanced distribution, with 53% experiencing moderate stress and equal proportions in low (22%) and high (25%) stress categories, likely due to structured curricula. These findings highlight the need for tailored support, particularly for Master’s students facing abrupt autonomy shifts and PhD candidates managing prolonged research demands.

**Table 7:** Stress level distribution by region and degree (Africa vs. Asia).

Region	Low stress	Moderate stress	High stress	Total
Africa	48 (17.9%)	139 (51.9%)	81 (30.2%)	268
Asia	62 (13.9%)	205 (46.0%)	179 (40.1%)	446
Degree				
Undergraduate	22.0	53.0	25.0	100.0
Master’s	15.0	48.0	37.0	100.0
PhD	20.0	35.0	45.0	100.0

This hierarchical linear regression Table 4 details the incremental contributions of demographic, academic, and social predictors to the variance in Stress, Anxiety, and Depression scores, with results standardized using beta coefficients (*β*), 95% confidence intervals (CIs), and False Discovery Rate-adjusted *p*-values (FDR-adj *p*). The analysis proceeds in three steps: first, demographics (Step 1) show that female gender is a significant positive predictor across all three outcomes, while age shows a non-significant negative trend, collectively explaining only 1.7–2.1% of the variance (*R*^2^). Second, adding academic factors (Step 2) substantially increases the explained variance (Δ*R*^2^ of 0.102–0.107), with PhD status and especially higher academic load emerging as strong, significant positive predictors. Finally, the inclusion of social factors (Step 3) yields a further significant increment (Δ*R*^2^ of 0.024–0.035), where both social isolation and language barrier are significant positive predictors, culminating in total models that explain 15.2–15.7% of the total variance in the mental health outcomes.

The correlation matrix in Table 5 reveals significant interrelationships between academic stressors and mental health indicators. Academic load demonstrates the strongest associations, with large effect size correlations to both stress (*r* = 0.58, *p* < 0.01) and anxiety (*r* = 0.53, *p* < 0.01). Language barriers and social isolation show moderate but meaningful relationships with mental health outcomes (range *r* = 0.27 to 0.42, all *p* < 0.01). The pattern of correlations suggests that while all measured stressors contribute to psychological distress, academic demands may be particularly salient. The robust correlation between stress and anxiety (*r* = 0.68, *p* < 0.01) confirms expected comorbidity between these mental health constructs. These findings align with existing literature on academic stress while highlighting the cumulative impact of multiple stressors on student wellbeing.

The most severe academic stressors included research/article burden (*M* = 4.5), thesis/dissertation pressure (*M* = 4.1), and heavy workload (*M* = 3.8), pointing to specific areas where institutional support such as structured research mentoring and workload caps could alleviate stress. Stress distribution varied by degree program, with master’s students reporting the highest levels of high stress (40%), compared to undergraduates and PhD students (25% each) as shown in Table 8. This pattern suggests that master’s programs, often involving a abrupt transition to independent research, may require additional mental health resources. In contrast, PhD students, despite their rigorous demands, might benefit from established support networks and longer adaptation periods.

**Table 8:** Top academic stressors (Mean scores, 1–5 scale).

Stressor	Mean (SD)	Rank
Research/Articles burden	4.5 (0.7)	1
Thesis/Dissertation	4.1 (0.8)	2
Heavy Workload	3.8 (0.9)	3
Language Barriers	3.6 (0.8)	4
Examination pressure	3.1 (0.7)	5

Table 9 presents a paradox in institutional support utilization. While thesis workshops show both high participation (65%) and satisfaction (*M* = 4.0, SD = 0.7), counseling services – arguably most critical for high-stress students – demonstrate both lower utilization (38%) and satisfaction (*M* = 3.0, SD = 1.3). The 20-percentage point gap between academic advising (58%) and counseling utilization may reflect either accessibility barriers or stigma surrounding mental health services. The notably higher standard deviation for counseling satisfaction (1.3 vs. 0.7–0.8 for other services) suggests particularly inconsistent experiences with mental health support.

**Table 9:** Utilization and satisfaction with support services.

Service	Mean satisfaction (SD)	% Using service
Counseling services	3.0 (1.3)	38.0
Academic advising	3.6 (0.8)	58.0
Thesis workshops	4.0 (0.7)	65.0

### 3.2 Qualitative insights from in-depth interviews

For conducting the qualitative data, the researcher included in-depth interviews from (*n* = 10) respondents. The analysis followed a consensual coding strategy to ensure rigor, culminating in the identification of core themes and sub-themes related to academic stress and mental health. The interviews were conducted in diverse languages including English, Urdu, and Sindhi for better understanding of the participants. Later the data was analyzed thematically using the process, translating, coding, decoding, finally the themes were made and interpreted in the Table 10. The results revealed that, international students studying aboard face significant academic pressures characterized by relentless workloads, fast-paced instruction, and language barriers. One of the respondent highlighted that, Studying abroad, particularly in China, presents significant challenges for international students. One major difficulty is the language barrier, as many scholars struggle to attain proficiency even after residing in the country for an extended period. For instance, some students report being unable to comprehend the local language even after a year, which hinders their academic and social integration. Many describe an unsustainable cycle of assignments, exams, and research demands, with one student stated that,

**Table 10:** The lived experience of international graduate student distress and resilience.

Theme	Sub-themes	Participant context
Academic stress	Overwhelming and cumulative workload	*When I complete one task, several more appear. It’s a cycle that never ends.*
	Pressure from research and publication	*My supervisor insisted I withdraw from a good Q2 journal to target only Q1. It doubled my stress and delayed everything.”*
Acculturative and institutional pressure	Language as a social barrier	*The long-lasting classes of about* 4 h*, heavy workload including reading articles for assignments and presentations increases the academic loads*
	Cultural distance and loneliness	Back home, my classmates and I would study together daily and share meals. Here, after 6 months, I still have not found that
	Mental health distresses	Waking up early in the morning, waiting for the busses to move to another campus has affected the mental health
Coping: between resilience and exhaustion	Reliance on peer networks	*My only saving grace is my group of friends from my country. We vent, we cook together, we understand each other without explaining*
	Emotional and physical depletion	*I am always tired. Even when I sleep, I’m thinking about my paper. Headaches are normal now. Sometimes I just feel completely empty*

"*When I complete the one task, several more appears*. Several more students included that “*academic pressure have affected the mental health of the students, besides that, we face stress including score, meetings as well as publications further contribute additionally.*

The quick tempo of lectures proves particularly challenging for those adjusting to a new education system, as falling behind in one concept can lead to prolonged difficulties. Even in English-medium programs, technical terminology and strong accents create additional obstacles to comprehension. These academic stressors contribute to serious mental health consequences. Nearly all participants reported chronic stress and anxiety, often accompanied by physical symptoms like sleep disturbances, appetite changes, and frequent headaches. Additionally, the combination of rigorous coursework and language difficulties has adversely affected candidature mental health. Miscommunication due to unfamiliar accents further complicates the learning process, exacerbating academic stress.

As, discussed by the students that “*Language barrier is one the biggest factor to adjust in the new environment”, secondly, the long lasting classes of about four hours, heavy work load including reading the articles for assignments and presentations increases the academic loads.*

Another contributing factor is the logistical challenges associated with attending classes on different campuses. The need to commute early in the morning disrupts students’ sleep schedules, leading to fatigue and heightened stress levels, which negatively impact both academic performance and mental health.

One of the student discussed that *‘I have been disturbed during the class schedules, as I need to wake up early in the morning and catch a bus to go for attending the class which is far away from the campus I already live. Besides that, even in the campus waking up early for the class is actually disturbing due to the sleep schedule.*

Emotional exhaustion is common, with students describing feeling completely drained and socially withdrawn. Many also experience imposter syndrome, doubting their abilities despite their academic accomplishments. Besides that, it was noticed that, the PhD candidates face additional pressures related to research and publication requirements. While targeting high-impact journals is a common expectation, the interference of supervisors in the publication process can be a significant source of stress. For example, one student reported being compelled to withdraw a manuscript from a Q2 journal which met their academic standards and resubmit to a Q1 journal at the insistence of their supervisor.

As depicted that “*I have written the paper targeting one specific journal who required the paper for special issue, while, as discussed by the supervisor that we need to target the high impact factor journal with the Q1 indexed in SSCI. Which has further increased the stress of work.*

Such demands not only increase workload but also contribute to mental strain, as students must navigate conflicting priorities between their own academic goals and the expectations of their advisors. While, the rejection of the paper broken my confidence and affected the mental health. Nevertheless, the culture do matters to mitigate or to increase the mental health, one of the respondent shared that,

*I used to communicate the lab mates but I totally ignore from them because of the language as well as culturally. Social isolation compounds these academic stresses. "Back home, my classmates and I would study together daily and share meals," shared a South Asian PhD candidate. "Here, after six months, I still eat alone in the cafeteria most days. While universities provide mental health services, significant barriers limit access”.*

Cultural stigma discourages help-seeking, language barriers restrict communication with predominantly Chinese-speaking counselors, and many students remain unaware of available support. Despite these institutional gaps, students employ various coping strategies. Peer networks offer vital emotional support, some faculty members provide unexpected accommodations, and self-care practices like exercise and meditation help manage stress. However, students emphasize that individual coping mechanisms cannot substitute for comprehensive institutional support tailored to international student’s mental health.

## 4 Discussion

The findings from this Nanjing-based study reveal significant mental health challenges among international students that both confirm and expand upon existing global research. Framed through an integrated theoretical lens combining Job Demands–Resources (JD-R) theory, Lazarus and Folkman’s Transactional Model of Stress and Coping, and acculturative stress frameworks our results elucidate how academic, social, and cultural demands interact to deplete psychological resources and overwhelm coping capacities. Our quantitative data shows alarmingly high stress levels, with 40% of participants reporting frequent stress symptoms - a figure notably higher than the 28–32% ranges found in comparable European studies (Li et al., 2020; Zieliński et al., 2024), this difference can be understood through JD-R theory. This disparity suggests the Chinese academic environment, with its unique blend of Confucian values and rapid internationalization, may create particularly intense pressures for foreign students. Several key findings stand out from our analysis. First, the hierarchical regression identified academic workload (*β* = 0.37, *p* < 0.001) as the strongest stress predictor, accounting for nearly 9% of variance in stress scores. This aligns with (Liao and Wei, 2014) work on acculturative stress but shows an even stronger effect size in the Nanjing context. From a transactional stress perspective, this suggests that academic demands are consistently appraised as threatening or exceeding coping resources, particularly among students without strong social or institutional buffers. Second, we found striking program-level differences, with master’s students reporting significantly higher stress (*β* = 0.24) than undergraduates – a pattern not seen in most Western studies. Qualitative interviews revealed this likely stems from the abrupt transition to independent research expectations combined with heavy coursework loads. Cultural factors emerged as critical mediators of stress experiences. Our correlation matrix showed language barriers (*r* = 0.42 with stress) and social isolation (*r* = 0.34) as significant contributors, while interviews revealed how Confucian classroom norms exacerbated these challenges. As one master’s student explained, “At home, professors encouraged questions, but here I feel I must remain silent.” This finding extends (Yu et al., 2014) work on Confucian educational values by showing their concrete impact on international student wellbeing. This imbalance creates a resource depletion cycle consistent with Conservation of Resources (COR) theory, wherein continuous loss of psychological, social, and academic resources without adequate replenishment leads to chronic stress and emotional exhaustion (Hobfoll, 1989).

The study’s mixed-methods approach proved particularly valuable in understanding service utilization patterns. While 65% of students used thesis workshops (with high satisfaction: *M* = 4.0), only 38% accessed counseling services (*M* = 3.0 satisfaction). Interview data suggests this stems from both cultural stigma and mismatched therapeutic approaches – issues also noted in Australian research (Chu et al., 2024) but appearing more pronounced in the Chinese context. From a resource-based perspective, thesis workshops represent a tangible, academically-legitimate resource, whereas counseling is often appraised through a cultural lens that associates help-seeking with weakness or loss of mianzi (face), thereby reducing its perceived utility as a coping resource. These findings have important implications for both theory and practice. They suggest that while some stressors are universal (e.g., academic pressure), their manifestation and intensity vary significantly across cultural contexts. This challenges one-size-fits-all approaches to international student support and argues for culturally grounded interventions. For Nanjing universities, our results indicate several promising directions: peer mentorship programs to address isolation, faculty training on inclusive pedagogy, and counseling services that incorporate diverse cultural perspectives on mental health. Future research should explore longitudinal patterns and compare different intervention models. As global higher education continues to internationalize, studies like this highlight the urgent need for institutions to adapt their support systems to diverse student populations’ needs while maintaining academic rigor.

## 5 Recommendations

To address the multifaceted challenges identified in this study, Nanjing universities should implement a comprehensive support framework that integrates academic, cultural, and mental health interventions. First, academic reforms should include workload adjustments for master’s programs, mandatory research mentoring, and transitional courses to bridge pedagogical gaps. Second, cultural adaptation initiatives must prioritize faculty training in inclusive teaching practices and establish peer mentorship programs to ease social integration. Third, mental health services require urgent enhancement through culturally sensitive counseling staffed by multilingual professionals and the embedding of support within academic departments to reduce stigma. Additionally, orientation programs should be restructured to provide realistic expectations about academic and cultural challenges while equipping students with stress-management tools. Finally, future research should evaluate the effectiveness of these interventions through longitudinal studies and cross-cultural comparisons. By adopting this holistic approach, institutions can foster an environment where international students thrive academically and personally, aligning with China’s broader goals for global higher education excellence.

### 5.1 Conclusion

The evidence presented in this study clearly demonstrates that overseas students in Nanjing must simultaneously cope with rigorous academic requirements and the difficulties of adapting to a foreign culture. Both numerical data and personal accounts consistently show that school-related pressures constitute the primary source of stress, though these effects are significantly shaped by cultural differences and the availability of institutional assistance. The acute stress levels among those pursuing master’s degrees indicate this academic stage demands particular consideration, just as the disparity between students’ psychological needs and current support offerings requires attention. These outcomes broaden international scholarship on student welfare by revealing how regional educational traditions modify common stressors into distinctive local variants. Ultimately, the research uncovers an important contradiction - Nanjing’s universities have attracted a diverse international student population but have not yet developed adequate systems to support their varied requirements as global scholars.

### 5.2 Study limitations

This research has several constraints that should be considered when interpreting the findings. The study’s cross-sectional nature means we cannot establish causal relationships between academic stressors and mental health outcomes. Self-reported measures may be subject to response biases, including participants’ tendency to underreport or over report symptoms due to social desirability, stigma, or cultural perceptions of mental health that discourage open disclosure. While the sample size was substantial, it primarily included students from African and Asian backgrounds at four Nanjing universities, potentially limiting the applicability of results to other regions or cultural groups. The qualitative interviews, though insightful, involved only 10 participants, restricting the diversity of perspectives captured. Additionally, the focus on Nanjing institutions may not fully represent international students’ experiences across China’s varied higher education landscape. These limitations highlight opportunities for future longitudinal studies with more diverse samples and multiple research sites to strengthen the validity and generalizability of findings.

## Data Availability

The datasets presented in this article are not readily available because the de-identified quantitative dataset, codebook, survey instruments, and qualitative interview guides (exact scale items) are available from the corresponding author upon reasonable request. Requests to access the datasets should be directed to lx20220614007@hhu.edu.cn.
