# Supplementary material for: The mental health crisis in global higher education: understanding and mitigating academic load stress among international students from Asia and Africa in Nanjing China
Source: Front Psychol. 2026 Jan 22;17:1707944. doi: 10.3389/fpsyg.2026.1707944 (PMC12873712; doi:10.3389/fpsyg.2026.1707944)
Supplement: Supplementary file 1 [file Table_1.DOCX]

| **S1. Qualitative Codebook and Data Presentation Matrix**  Supplementary Table S-1 provides a verifiable overview of the final thematic structure. It includes the core themes and sub-themes derived from the consensual analysis, along with illustrative quotations (de-identified) to demonstrate the basis for each theme. This matrix allows readers to triangulate the interpreted results with raw data excerpts. | | | | |
| --- | --- | --- | --- | --- |
| **Main Theme** | **Sub-theme** | **Operational Definition** | **Representative Quotation 1** | **Representative Quotation 2** |
| **1. Academic Stress** | 1.1 Overwhelming & Cumulative Workload | Perceptions of academic tasks as incessant, excessive, and unmanageable in volume or pace. | *"When I complete one task, several more appear. It's a cycle that never ends."* (Participant 04, Master's) | *"The deadlines are relentless. You submit one paper and the next assignment is already overdue in your mind."* (Participant 07, PhD) |
|  | 1.2 Pressure from Research & Publication | Stress specifically stemming from thesis requirements, journal submissions, and supervisor expectations regarding publication quality/quantity. | *"My supervisor insisted I withdraw from a good Q2 journal to target only Q1. It doubled my stress and delayed everything."* (Participant 02, PhD) | *"The 'publish or perish' mentality here is palpable. It's not just about finishing the degree, but where you publish."* (Participant 09, PhD) |
| **2. Acculturative & Institutional Pressure** | 2.1 Language as an Academic Barrier | Challenges in comprehension, expression, or academic writing in Chinese or English that directly hinder learning and performance. | *"The long classes... heavy workload including reading articles for assignments and presentations increases the academic loads, and if you don't understand the language fully, it's paralyzing."* (Participant 05, Master's) | *"Even in English-taught programs, the technical vocabulary and accents make some lectures very difficult to follow."* (Participant 10, Undergraduate) |
|  | 2.2 Cultural Distance & Loneliness | Feelings of social isolation stemming from differences in social norms, communication styles, and the absence of familiar community. | *"Back home, my classmates and I would study together daily and share meals. Here, after six months, I still haven't found that."* (Participant 03, PhD) | *"I used to communicate with lab mates but I totally ignore them because of the language as well as cultural differences. It's lonely."* (Participant 06, Master's) |
| **3. Coping: Between Resilience & Exhaustion** | 3.1 Reliance on Peer Networks | Turning to friends, especially from the same cultural/linguistic background, for emotional support, practical help, and a sense of belonging. | *"My only saving grace is my group of friends from my country. We vent, we cook together, we understand each other without explaining."* (Participant 01, Master's) | *"We have a WhatsApp group. Whenever someone is stressed about a deadline, we all chime in with support. It's our survival network."* (Participant 08, Undergraduate) |
|  | 3.2 Emotional & Physical Depletion | Manifestations of chronic stress in the form of mental fatigue, emotional numbness, and somatic symptoms. | *"I am always tired. Even when I sleep, I'm thinking about my paper. Headaches are normal now. Sometimes I just feel completely empty."* (Participant 05, Master's) | *"The constant anxiety has affected my sleep and appetite. I feel drained before the day even starts."* (Participant 07, PhD) |
