# Supplementary material for: The mental health crisis in global higher education: understanding and mitigating academic load stress among international students from Asia and Africa in Nanjing China
Source: Front Psychol. 2026 Jan 22;17:1707944. doi: 10.3389/fpsyg.2026.1707944 (PMC12873712; doi:10.3389/fpsyg.2026.1707944)
Supplement: Supplementary file 2 [file Table_2.DOCX]

| **S-2: Table 2: Item-Level Standardized Factor Loadings, Average Variance Extracted (AVE), and Composite Reliability (CR) for Latent Constructs** | | | | |
| --- | --- | --- | --- | --- |
| **Construct** | **Item Code** | **Standardized Loading (λ)** | **AVE** | **CR** |
| **Anxiety** | ANX1 | 0.72 | 0.45 | 0.88 |
|  | ANX2 | 0.69 |  |  |
|  | ANX3 | 0.71 |  |  |
|  | ANX4 | 0.71 |  |  |
|  | ANX5 | 0.70 |  |  |
|  | ANX6 | 0.72 |  |  |
|  | ANX7 | 0.70 |  |  |
| **Academic Stress** | ACS1 | 0.78 | 0.53 | 0.91 |
|  | ACS2 | 0.75 |  |  |
|  | ACS3 | 0.81 |  |  |
|  | ACS4 | 0.70 |  |  |
|  | ACS5 | 0.72 |  |  |
|  | ACS6 | 0.74 |  |  |
|  | ACS7 | 0.73 |  |  |
| **Depression** | DEP1 | 0.74 | 0.51 | 0.80 |
|  | DEP2 | 0.71 |  |  |
|  | DEP3 | 0.77 |  |  |
|  | DEP4 | 0.70 |  |  |
|  | DEP5 | 0.70 |  |  |
|  | DEP6 | 0.73 |  |  |
|  | DEP7 | 0.71 |  |  |
| **Support Services** | SUP1 | 0.82 | 0.56 | 0.79 |
|  | SUP2 | 0.75 |  |  |
|  | SUP3 | 0.70 |  |  |
