# Supplementary material for: The mental health crisis in global higher education: understanding and mitigating academic load stress among international students from Asia and Africa in Nanjing China
Source: Front Psychol. 2026 Jan 22;17:1707944. doi: 10.3389/fpsyg.2026.1707944 (PMC12873712; doi:10.3389/fpsyg.2026.1707944)
Supplement: Supplementary file 3 [file Table_3.DOCX]

| Construct | Item Code | Item Description (Abbreviated) | Loading | SE | p-value |
| --- | --- | --- | --- | --- | --- |
| **Anxiety** | ANX1 | Feeling nervous or anxious | 0.72 | 0.04 | <.001 |
|  | ANX2 | Unable to control worrying | 0.68 | 0.04 | <.001 |
|  | ANX3 | Worrying too much | 0.75 | 0.03 | <.001 |
|  | ANX4 | Trouble relaxing | 0.70 | 0.04 | <.001 |
|  | ANX5 | Restlessness | 0.65 | 0.05 | <.001 |
|  | ANX6 | Easily annoyed | 0.71 | 0.04 | <.001 |
|  | ANX7 | Feeling afraid | 0.69 | 0.04 | <.001 |
| **Academic Stress** | STR1 | Academic workload | 0.78 | 0.03 | <.001 |
|  | STR2 | Deadline pressure | 0.74 | 0.03 | <.001 |
|  | STR3 | Difficulty with coursework | 0.80 | 0.03 | <.001 |
|  | STR4 | Performance expectations | 0.76 | 0.03 | <.001 |
|  | STR5 | Exam preparation stress | 0.72 | 0.04 | <.001 |
|  | STR6 | Thesis/dissertation pressure | 0.79 | 0.03 | <.001 |
|  | STR7 | Publication demands | 0.75 | 0.03 | <.001 |
| **Depression** | DEP1 | Feeling down or hopeless | 0.73 | 0.04 | <.001 |
|  | DEP2 | Loss of interest in activities | 0.70 | 0.04 | <.001 |
|  | DEP3 | Trouble sleeping | 0.76 | 0.03 | <.001 |
|  | DEP4 | Feeling tired | 0.71 | 0.04 | <.001 |
|  | DEP5 | Poor appetite | 0.68 | 0.05 | <.001 |
|  | DEP6 | Feeling bad about self | 0.74 | 0.04 | <.001 |
|  | DEP7 | Trouble concentrating | 0.69 | 0.04 | <.001 |
| **Support Services** | SUP1 | Availability of counseling | 0.82 | 0.03 | <.001 |
|  | SUP2 | Helpfulness of academic advising | 0.79 | 0.03 | <.001 |
|  | SUP3 | Usefulness of workshops | 0.85 | 0.03 | <.001 |

Supplementary Material

**S-3: Table-3: Standardized Factor Loadings from Confirmatory Factor Analysis (N = 1,115)**
